# Astragaloside IV alleviates stroke-triggered early brain injury by modulating neuroinflammation and ferroptosis via the Nrf2/HO-1 signaling pathway

**DOI:** 10.1590/acb380723

**Published:** 2023-03-24

**Authors:** Chunlei Zhang, Zhonghua Shi, Qinyi Xu, Jianqing He, Lei Chen, Zehua Lu, Qiaohua Huan, Yuhai Wang, Gang Cui

**Affiliations:** 1 Soochow University – First Affiliated Hospital – Department of Neurosurgery – Jiangsu, China.; 2 Anhui Medical University – Wuxi Clinical College – 904th Hospital of Joint Logistic Support Force of PLA – Department of Neurosurgery – Wuxi, China.; 3 Wuxi Huishan Peoples Hospital – Department of Neurosurgery – Jiangsu, China.; 4 904th Hospital of Joint Logistic Support Force of PLA – Department of Radiology – Jiangsu, China.

**Keywords:** Brain Injuries, Neuroinflammatory Diseases, Ferroptosis, Stroke

## Abstract

**Purpose:**

Stroke is an acute cerebrovascular disease. Astragaloside IV (AS-IV) is an active ingredient extracted from *Astragalus membranaceus* with an established therapeutic effect on central nervous system diseases. This study examined the neuroprotective properties and possible mechanisms of AS-IV in stroke-triggered early brain injury (EBI) in a rat transient middle cerebral artery occlusion (MCAO) model.

**Methods:**

The neurological scores and brain water content were analyzed. 2,3,5-triphenyl tetrazolium chloride (TTC) staining was utilized to determine the infarct volume, neuroinflammatory cytokine levels, and ferroptosis-related genes and proteins, and neuronal damage and molecular mechanisms were evaluated by terminal deoxynucleotidyl transferase dutp nick-end labeling (TUNEL) staining, western blotting, and real-time polymerase chain reaction.

**Results:**

AS-IV administration decreased the infarct volume, brain edema, neurological deficits, and inflammatory cytokines TNF-α, interleukin-1β (IL-1β), IL-6, and NF-κB, increased the levels of SLC7A11 and glutathione peroxidase 4 (GPX4), decreased lipid reactive oxygen species (ROS) levels, and prevented neuronal ferroptosis. Meanwhile, AS-IV triggered the Nrf2/HO-1 signaling pathway and alleviated ferroptosis due to the induction of stroke.

**Conclusions:**

Hence, the findings of this research illustrate that AS-IV administration can improve delayed ischemic neurological deficits and decrease neuronal death by modulating nuroinflammation and ferroptosis via the Nrf2/HO-1 signaling pathway.

## Introduction

Ischemic stroke is a kind of acute cerebrovascular illness that is a major contributing factor to long-term impairment in industrialized nations and one of the major contributors to death around the globe^1^. The GBD 2019 Stroke Collaborators reported that there were 101 million prevalent strokes, 12.2 million incident stroke attacks, 143 million disability-adjusted life years attributed to stroke, and 6.55 million deaths from stroke in 2019, a higher incidence and the first leading cause of death in China^2^^,^ 3. The early intervention approach to cerebral ischemia focuses on the restoration of cerebral blood flow and reoxygenation, which is used for salvaging cells in the penumbra region and reducing the infarct volume as soon as possible^4^^,^ 5. However, an increasing number of studies have shown that the accumulation of oxidative free radicals can result from reperfusion injury after cerebral blood reperfusion and the restoration of oxygen-rich blood, which is ischemia-reperfusion (I/R) injury^6^^–^ 10.

The pathophysiology of cerebral I/R injury is quite complex and is characterized by the overproduction of reactive oxygen species (ROS), dramatically increased release of extracellular excitatory amino acids and glutamate levels, and activation of autophagy, apoptosis, ferroptosis, necrosis, endoplasmic reticulum (ER) stress, oxidative stress, and neuroinflammation^4^^,^ 6 ^–^ 14. In particular, ROS and neuroinflammation perform instrumental functions in cerebral I/R injury^6^^,^ 13 ^–^ 15. ROS overproduction and excessive accumulation of inflammatory factors lead to neuronal apoptosis and ferroptosis^11^^,^ 15 ^–^ 17. According to previous studies^17^^–^ 21, inhibition of ferroptosis and neuroinflammation can improve neurological deficits and alleviate cerebral edema after cerebral I/R injury in animals. Therefore, therapeutic strategies to inhibit ferroptosis, decrease neuroinflammation, and block the generation of ROS have important clinical application value and could be used to improve cerebral I/R injury. However, no safe and clinically effective treatment methods or drugs can prevent and improve cerebral I/R injury.

**Figure 1:**
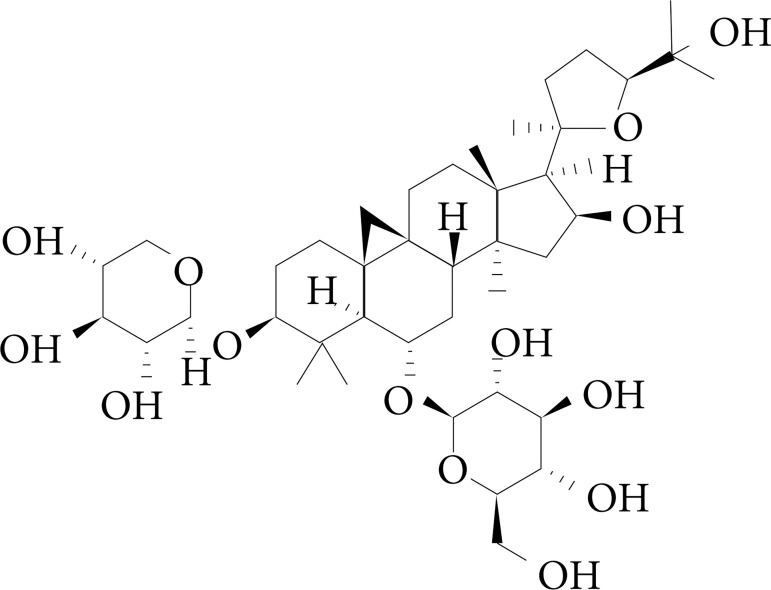
The chemical structure of astragaloside IV.

Many natural compounds have been reported to exhibit therapeutic functions by modulating ferroptosis^22^^,^ 23. Astragaloside IV (AS-IV), a newly discovered glycoside of cyclobutane-type triterpene extracts from *Astragalus membranaceus*, has proven to be effective (Fig. 1)^24^. AS-IV is effective in treating several neurodegenerative disorders, including Alzheimer’s disease, Parkinson’s disease, and cerebral ischemia^25^^–^ 29. It is intriguing to note that rats treated with AS-IV for 24 h showed significant improvement in learning, memory, motor status, and neurological function. Our recent study also demonstrated that AS-IV inhibits the process of ferroptosis in SAH by activating the Nrf2/HO-1 pathway^30^.

Cerebral I/R injury develops from ROS, an inflammatory cytokine, and glutamate-induced excitotoxicity, which can lead to rapid neuronal death in the brain^20^^,^ 31. The molecular signaling pathways of neuroinflammation and ferroptosis are complex. Activation of Nrf2 has been reported to occur in many diseases, with abnormal expression after ferroptosis and amino acid depletion^32^^–^ 34. Whether AS-IV alleviates cerebral I/R damage caused by regulating neuroinflammation and ferroptosis through the Nrf2/HO-1 signaling pathway remains unclear. Hence, we constructed a rat cerebral I/R injury model to explore the neuroprotection of AS-IV and studied the interplay between neuroinflammation and ferroptosis.

## Methods

### Animals

All experimental tests were carried out on healthy Sprague-Dawley rats (8–10 weeks old, Nantong University, Nantong, China). A total of 45 rats were used and randomly assigned to the sham, middle cerebral artery occlusion (MCAO), and MCAO+AS-IV groups. Each of the animal tests conducted for this research followed the National Institutes of Health criteria for the care and use of laboratory animals and was approved by the Ethics Committee of the 904^th^ Hospital of Joint Logistic Support Force of PLA (YXLL-2022011).

### Transient MCAO animal model

Following the intraluminal filament approach, the rat MCAO model was produced in full compliance with a previously reported procedure^35^. Afterward, 1% sodium pentobarbital (40 mg/kg) was administered to the rats intraperitoneally to anesthetize them before placing them in a brain stereotaxic device. An incision was created in the midline of the neck to expose the common internal and external carotid arteries. After ligating and cutting the external carotid artery on the left side, a 3-mm stump was exposed. We then perforated the carotid artery at the bifurcation of the middle and anterior cerebral arteries utilizing an 18–20-mm-long surgical filament (0.26 mm diameter; Beijing Cinontech Co. Ltd., China) was threaded through the external carotid artery stump into the internal carotid artery and left in situ for 120 min. After that, the filament was withdrawn to facilitate reperfusion. Rats in the sham surgery group received the identical procedure as the other rats but without filament insertion.

### Preparation and administration of drugs

Following the successful establishment of the MCAO model, AS-IV (No. 84687–43-4, purity ≥ 98%) was obtained from Nanjing Spring and Autumn Biotech Co., Ltd. (Nanjing, China). The injection of AS-IV (20 mg/kg) was carried out intraperitoneally (i.p.) at 30 min after MCAO induction.

### Assessment of neurobehavioral functioning

Neurological functionality was measured 72 h following MCAO establishment to determine the degree of brain damage, as reported earlier^36^. Each rat belonging to each group was subjected to a behavioral evaluation, with a greater score indicating enhanced neurological function.

### Determination of the water content in the brain

The degree of cerebral edema was ascertained by assessing the content of water in the brain utilizing the conventional wet-dry technique, as described earlier^36^^–^ 38. It was decided that the rats should be euthanized 72 h following MCAO, and the whole brain was extracted (wet weight). Brain samples from rats in each group were subsequently dried for 24 h at 105 °C to obtain dry weights. The proportion of the water content in the brain was equivalent to (wet weight – dry weight) / wet weight × 100%.

### 2,3,5-triphenyl tetrazolium chloride (TTC) staining and infarct volume measurement

By using TTC (Sigma, T8877), we successfully determined the volume of the infarct at 72 h following transient MCAO. The procedure protocol was conducted as previously reported^39^. Briefly, fresh rat brain samples were collected after the rats were sacrificed and then rinsed using ice-cold phosphate-buffered saline. After this, the brain was kept at –20 °C for 15 min before being sliced into 2-mm coronal slices utilizing a brain mold in preparation for the next steps. We then stained the brain slices with 2% TTC solution for 20 min at 37 °C in the dark. The viable portion of the brain segment was dyed red, whereas the dead portion was dyed pale white. Images of these segments were taken, and the infarct volume was computed with ImageJ.

### Measurements of cytokines in ipsilateral cortical tissue

The ELISA kit was utilized following the guidelines stipulated by the manufacturer to determine the levels of cerebral cortex NF-κB (cat. no. ab176663; Abcam), TNF-α (cat. no. ab208348; Abcam), IL-6 (cat. no. ab222503; Abcam), and IL-1β (cat. no. ab197742; Abcam).

### Terminal deoxynucleotidyl transferase dutp nick-end labeling (TUNEL) staining

To determine neuronal cell death in the cerebral cortex, a TUNEL test was utilized. Each sample was introduced into a 50 μL TUNEL reaction solution, and the slides were subjected to incubation for 60 min at 37 °C in a humid darkened chamber; 4’,6-diamidino-2-phenylindole was subsequently applied to the slides for 5 min at ambient temperature in the dark to stain the nuclei, after which the slides were photographed with a fluorescence microscope. The process, which used a TUNEL staining kit, was carried out in compliance with the package recommendations. A negative control (i.e., one that did not include any of the TUNEL reaction media) was employed. The cell density was checked in 4 high-power fields that were chosen at random, and the collected data from each field were combined to compute the average value.

### Measurements of malondialdehyde (MDA) and glutathione (GSH) contents

Levels of MDA and GSH in cerebral cortex tissue were investigated by using commercialized assay kits (Beyotime, China) 72 h after MCAO, following the manufacturer’s instructions. The absorbance of MDA in the samples was detected at 532 nm, and the concentration of GSH was determined at 412 nm.

### Lipid ROS measurement

ROS levels in cerebral cortex tissue were detected using a ROS kit (Beyotime, China). Briefly, brain tissue samples were homogenized and centrifuged at 10,000 g and 4 °C for 15 min. ROS levels in tissues were detected using DCFH-DA according to the manufacturer’s instructions. Fluorescence intensity was detected using a fluorescence microplate reader (Molecular Devices, United States) with an excitation wavelength of 485 nm and an emission wavelength of 530 nm. All data were normalized to the Sham group.

### Western blotting

Western blot analyses were carried out in the same manner as reported earlier^36^. Samples of the cerebral cortex were harvested, homogenized, and isolated utilizing sodium dodecyl sulfate-polyacrylamide gel electrophoresis on 10% polyacrylamide gels. To assess protein content, we employed a BCA Protein Assay Kit (Beyotime) using the bicinchoninic acid technique. Upon isolation, the protein specimens were subsequently placed onto Immobilon nitrocellulose membranes. Blocking of the membranes was performed for 1 h at ambient temperature using 5% nonfat milk. After that, the membranes were treated using the primary antibodies listed below for an overnight period at 4 °C: rabbit anti-β-actin (1:1,000, mouse monoclonal, Abcam, ab8226), rabbit anti-Nrf2 (1:1,000, Abcam, ab92946), rabbit anti-glutathione peroxidase 4 (GPX4) (1:500, cat# A1933, Abclonal), rabbit anti-SLC7A11 (1:500, cat# A15604; Abclonal), and rabbit anti-HO-1 (1:1,000, Abcam, ab186284). Once the membranes had been rinsed three times using TBST, they were subjected to a 1.5-h incubation at ambient temperature with secondary antibodies, including goat anti-mouse IgG secondary antibodies or HRP-conjugated goat anti-rabbit IgG (1:5,000). A Bio-Rad imaging system (Bio-Rad, Hercules, CA, USA) was utilized for the detection of the protein bands, which were subsequently quantified utilizing ImageJ software.

### Quantitative real-time polymerase chain reaction (qPCR)

Quantitative real-time PCR analysis was performed as previously indicated. Total RNA was extracted from cerebral cortex samples using TRIzol Reagent (Gibco; Thermo Fisher Scientific, Inc., Waltham, MA, USA) according to the manufacturer’s instructions. Then, RNA was reverse transcribed to complementary DNA (cDNA) using the RevertAid First Strand cDNA Synthesis Kit (K1622; Thermo Fisher Scientific Inc., Rockford, IL). The HO-1 and Nrf2 mRNA levels in each sample were measured by qPCR using SYBR Green Master Mix (Toyobo Co., Ltd., Osaka, Japan). GAPDH was used as an internal control. The qPCR thermocycling conditions were as follows: 45 °C (2 min) and 95 °C (10 min), followed by 40 cycles of denaturation at 95 °C (15 s), annealing at 60 °C (1 min), and extension at 72 °C (1 min). All samples were analyzed in triplicate. The target genes and the specific primers are as follows:

HO-1 (forward, 5’-TGACAGAAGAGGCTAAGACCG-3’; reverse, 5’-AGTGAGGACCCACTGGAGGA-3’),

Nrf2 (forward, 5‘-CAGTGCTCCTATGCGTGAA-3’; reverse, 5‘-GCGGCTTGAATGTTTGTCT-3’)

GAPDH (forward, 5’- ATGGGTGTGAACCACGAGA-3’ and reverse, 5’-CAGGGATGATGTTCTGGGCA-3’)

### Statistical analysis

All experimental tests were replicated three times, and the data are presented as the means and standard deviation (SD). Analyses of all statistical data were accomplished utilizing GraphPad Prism 6 (GraphPad Software, San Diego, CA, USA) and SPSS 14.0 (SPSS, Chicago, IL, USA). In the case of two groups being compared, Student’s t-test was employed, and in the case of two independent variables being compared, a one-way analysis of variance (ANOVA) accompanied by Bonferroni’s post-hoc test was utilized. Specifically, we executed the Kruskal–Wallis test, accompanied by Dunn’s post-hoc test, to analyze the data with nonnormal distribution and/or nonhomogeneous variance. Statistical significance for all the statistical data was established at p < 0.05.

## Results

### AS-IV alleviates cerebral I/R injury and brain edema after MCAO

**Figure 2:**
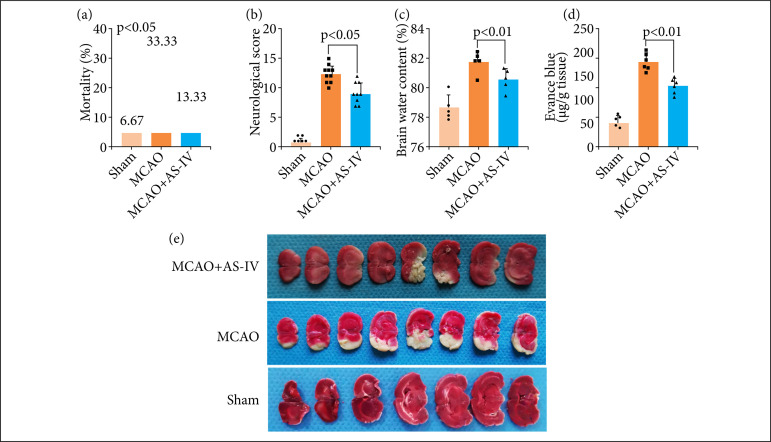
AS-IV alleviates cerebral I/R injury and brain edema after MCAO. **(a)** The death rates were elevated in the MCAO group and decreased following AS-IV therapy. **(b)** Neurological scores of rats in the three groups at 72 h following MCAO. **(c)** AS-IV alleviates the water content in the brain after MCAO. **(d)** AS-IV alleviates BBB permeability after MCAO. **(e)** Representative photograph of rat coronal sections stained with TTC at 72 h after MCAO. MCAO: middle cerebral artery occlusion; AS-IV: astragaloside IV. Data are represented as the mean ± SD.

We developed the MCAO model and administered AS-IV following cerebral I/R damage. The impact of AS-IV therapies on long-term neurological impairment metrics, such as death rates and neurological scores, was investigated in this study. As depicted in Fig. 2, a decrease in the mortality rates (Fig. 2a) was observed in the MCAO+ AS-IV groups, although there were no remarkable differences in contrast with the MCAO group (p > 0.05). A remarkable decrease in neurological scores was discovered following MCAO, and AS-IV administration remarkably elevated neurological scores (p < 0.05, Fig. 2b). To clarify the EBI and cerebral I/R injury after MCAO, we measured the water content in the brain utilizing the wet–dry technique for 72 h following MCAO to assess the extent of brain damage. The findings illustrated a substantial elevation in the water content of the brain following MCAO, which was alleviated after AS-IV administration (Fig. 2c). Comparable findings were recorded for blood brain barrier (BBB) permeability, which was shown to be considerably increased following MCAO, and AS-IV treatment remarkably ameliorated this permeability (Fig. 2d). Additionally, compared to the sham operation group, the MCAO group exhibited a greater cerebral infarct size, while the cerebral infarction area was significantly improved in the AS-IV cohort (Fig. 2e).

### AS-IV decreased neuronal damage and ROS levels after MCAO

Neuronal damage is the main factor that leads to cerebral I/R injury after MCAO. Therefore, the degree of cell death in MCAO rats subjected to treatment with and without AS-IV at 72 h following model formation was assessed utilizing a TUNEL test. The TUNEL staining findings demonstrated more hippocampal neuronal death following MCAO, which was attenuated by AS-IV (Fig. 3a;b). Lipid ROS measurement showed that lipid ROS levels increased in MCAO rats and decreased significantly after AS-IV treatment (Fig. 3c).

**OFigure 3:**
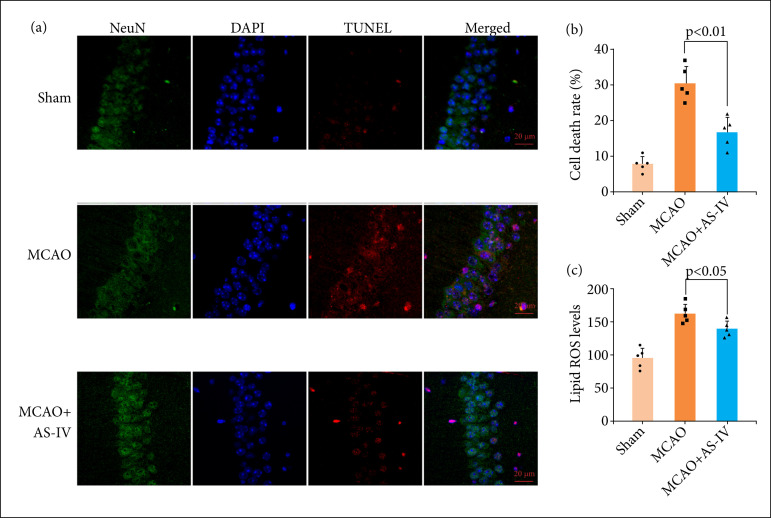
AS-IV decreased neuronal damage and ROS levels after MCAO. **(a)** TUNEL staining showed that AS-IV attenuated cell death in the hippocampus 72 h following MCAO. **(b)** Quantitative measurement of TUNEL staining. **(c)** Quantitative analysis of lipid ROS. Scale bar = 50 μm. TUNEL: terminal deoxynucleotidyl transferase dUTP nick end labeling; DAPI: 4’,6-diamidino-2-phenylindole. Data are represented as the mean ± SD.

### AS-IV ameliorates neuroinflammation after MCAO

**Figure 4:**
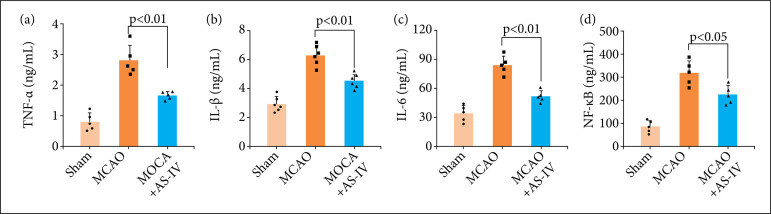
AS-IV ameliorates neuroinflammation after MCAO. AS-IV remarkably lowered the levels of hippocampal **(a)** TNF-α, **(b)** IL-1β, **(c)** IL-6, and **(d)** NF-κB at 72 h following MCAO. Data are represented as the mean ± SD.

As discovered in earlier studies, neuroinflammation performs a critical function in the onset and progression of cerebral I/R damage after MCAO^40^. The inflammatory complex triggers the release of proinflammatory cytokines, such as TNF-α, IL-6, and IL-1β, and the consequent stimulation of proinflammatory signaling via NF-κB. Therefore, we measured the hippocampal expression levels of TNF-α, NF-κB, IL-6, and IL-1β utilizing ELISAs. The expression levels of proinflammatory cytokines were considerably enhanced after MCAO, whereas those of proinflammatory cytokines were dramatically attenuated following AS-IV administration (Fig. 4a-d). These findings illustrated that AS-IV exerted a substantial anti-inflammatory effect against the MCAO-triggered neuroinflammatory response.

### AS-IV ameliorates ferroptosis after MCAO

**Figure 5:**
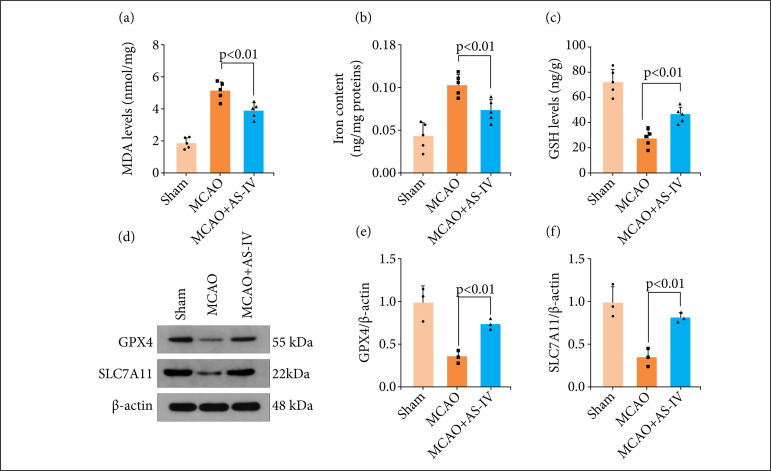
AS-IV ameliorates ferroptosis after MCAO. AS-IV remarkably lowered the levels of hippocampal **(a)** MDA, **(b)** iron, and **(c)** GSH. (d, e) Western blot analysis of the protein expression levels of SLC7A11 and GPX4. Data are represented as the mean ± SD.

The previous detection confirmed that lipid ROS levels increased in MCAO rats and decreased significantly after AS-IV treatment (Fig. 3c). Further studies showed that the levels of MDA (Fig. 5a) and iron content (Fig. 5b) increased significantly after MCAO, and the levels of GSH (Fig. 5c) decreased significantly after MCAO, which was reversed after AS-IV administration. Therefore, AS-IV treatment appears to reduce iron content and alleviate lipid peroxidation in rat brains after MCAO.Western blot analysis of the expression of ferroptosis-associated proteins after MCAO was utilized to assess the influence of AS-IV on ferroptosis (Fig. 5d). The findings of the western blotting assay also revealed that AS-IV can lower the levels of the ferroptosis-associated proteins GPX4 and SLC7A11 (Fig. 5e; f). Hence, we postulated that the neuroprotective properties of AS-IV are attributed to its ability to suppress ferroptosis.

### AS-IV regulates neuroinflammation and ferroptosis by modulating the Nrf2/HO-1 signaling pathway after MCAO

**Figure 6:**
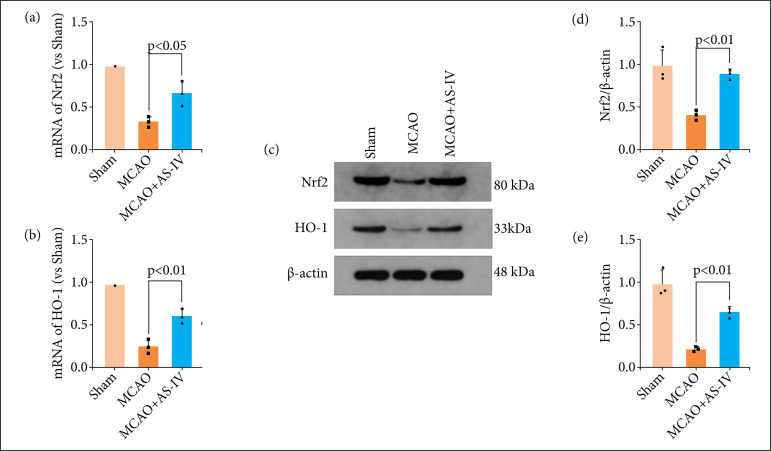
AS-IV regulates neuroinflammation and ferroptosis by modulating the Nrf2/HO-1 signaling pathway after MCAO. Nrf2 and HO-1 in the rat brain cortex after MCAO were evaluated by reverse transcription-qPCR. MCAO markedly enhanced Nrf2 **(a)** and HO-1 **(b)** mRNA expression, while this trend was reversed after AS-IV treatment. **(c)** Western blotting was utilized to determine the protein expression levels of Nrf2 and HO-1 in the rat cerebral cortex after MCAO. AS-IV markedly increased the protein expression levels of **(d)** Nrf2 and **(e)** HO-1 after MCAO. Data are represented as the mean ± SD.

To explore the molecular mechanism whereby AS-IV alleviates early brain injury (EBI) after MCAO, we examined the key members in the Nrf2/HO-1 signaling pathway. Therefore, we detected the protein and gene expression profiles of Nrf2 and HO-1 after MCAO and AS-IV induction by RT-qPCR and western blotting. The findings from RT-qPCR illustrated that the levels of Nrf2 and HO-1 were elevated following MCAO and alleviated after AS-IV treatment (Fig. 6a; b). Comparable findings were recorded by western blotting analysis of the levels of protein expression (Fig. 6c-e). Therefore, it was postulated that AS-IV suppresses EBI by suppressing the Nrf2/HO-1 signaling pathway after MCAO.

### Nrf2/HO-1 inhibitor reversed the neuroprotection of AS-IV after MCAO

**Figure 7:**
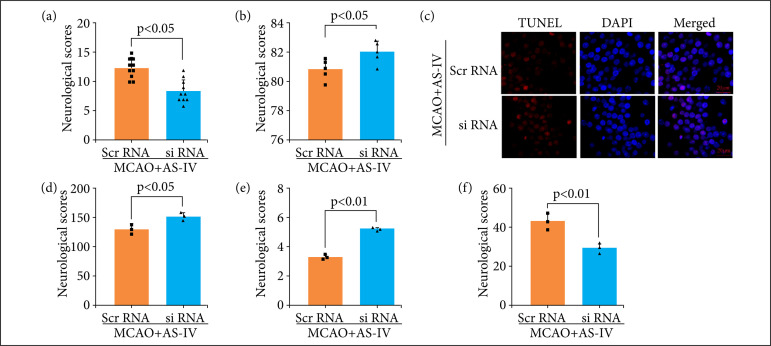
Nrf2/HO-1 inhibitor reversed the neuroprotection of AS-IV after MCAO. Knockdown of Nrf2 reversed the neuroprotective effect of AS-IV at 72 h post MCAO. (a, b) Neurological scores and brain water content 72 h after MCAO in rats. **(c)** TUNEL staining. Knockdown of Nrf2 enhanced cell death in the hippocampus 72 h after MCAO. (d, f) Quantitative analysis of lipid ROS, MDA, and GSH levels. Data are represented as the mean ± SD.

Nrf2-targeting siRNA was used to knock down the expression of Nrf2 in the animal MCAO model, which was procured from GeneChem (Shanghai, China), to investigate the neuroprotective effects of AS-IV following Nrf2 downregulation. The neurological scores decreased significantly after the Nrf2 knockdown (Fig. 7a), and the brain water content increased significantly after the Nrf2 knockdown (Fig. 7b). TUNEL staining also showed that Nrf2 knockdown led to significantly aggravated neuronal death (Fig. 7c). These data showed that Nrf2 knockdown reversed the neuroprotection of AS-IV after MCAO. Lipid ROS (Fig. 7d) and MDA (Fig. 7e) levels in the MCAO + AS-IV + siRNA group were significantly higher than those in the MCAO + AS-IV + scr-RNA group. The GSH (Fig. 7f) level also decreased significantly after the Nrf2 knockdown. Hence, the anti-ferroptosis effect was blocked after the Nrf2 knockdown.

## Discussion

In the present study, we examined the therapeutic roles of AS-IV in mitigating EBI in a rat model of MCAO. As illustrated in this work, AS-IV plays a neuroprotective role that helps to reduce the severity of EBI after MCAO. We discovered that AS-IV (1) improves neurological dysfunction after MCAO, (2) alleviates neuronal damage in *vitro*, (3) relieves neuroinflammation and prevents ferroptosis following MCAO, and (4) the anti-ferroptosis and anti-neuroinflammation properties of AS-IV might be attributed to the Nrf2/HO-1 signaling pathway.

AS-IV is a primary active ingredient of the Chinese herb Radix Astragali, which protects against acute cerebral ischemic/reperfusion/hemorrhagic injury through its antioxidant, anti-inflammatory, and antiapoptotic properties^30^^,^ 41 ^,^ 42. In a rat MCAO model, AS-IV relieved cerebral ischemia/reperfusion injury and enhanced neurogenesis, angiogenesis, and neurological functional recovery partially by transforming microglia/macrophages from the M1 to the M2 phenotype in a PPARγ-dependent manner^41^. Liu et al.^42^ also reported that AS-IV protected the integrity of the BBB in LPS-induced mice, the mechanism of which might be mediated by activating the Nrf2 signaling pathway. Additionally, AS-IV can attenuate neurological deficits in rats with I/R injury and decrease cerebral infarction and neuronal apoptosis by inhibiting the activation of key factors in the death receptor pathway and mitochondrial pathway^44^. In the present study, we also found that AS-IV can improve delayed ischemic neurological deficits, reduce cerebral infarction, and decrease neuronal death by modulating neuroinflammation and ferroptosis. To our knowledge, this was the first study to explore the anti-ferroptosis effects of AS-IV after I/R injury.

Ferroptosis is a nonapoptotic form of iron-dependent programmed cell death that differs from traditional cell death processes, such as apoptosis and autophagy, and is mostly caused by a disturbance of iron homeostasis and accumulation of lipid ROS in the cytoplasm^11^. The classical morphological characteristics of cell death are the disappearance of mitochondrial cristae and significantly narrowed mitochondria, thickening of the lipid bilayer membrane, small cell size, and reduced cell connections that lead to cell separation^11^^,^ 45. Ahmad et al.^46^ reported that sesamin induces significant neuroprotection by ameliorating many signaling pathways; the level of GSH was markedly reduced and lipid peroxidation increased after ischemic stroke and exhibited an effect similar to that in PC-12 cells in an oxygen-glucose deprivation (OGD) experimental model^47^. Alim et al.^48^ neuronal ferroptosis plays an important role in ischemic stroke, and systemic administration of a brain-penetrant selenopeptide activates homeostatic transcription to inhibit cell death and improve function after ischemic stroke. Recent studies have also demonstrated that inhibiting ACSL4 can promote the recovery of neurological function after stroke by suppressing ferroptosis^49^^-^ 51.

The mechanisms and molecules regulating neuroinflammation and ferroptosis are very complex. According to the findings from the current research, AS-IV decreases the levels of hippocampal NF-κB, TNF-α, IL-6, and IL-1β production, subsequently inhibiting neuroinflammation, and AS-IV can also decrease the levels of ferroptosis. Additionally, we found that the antineuroinflammatory and anti-ferroptosis impacts of AS-IV might be linked to the Nrf2/HO-1 signaling pathway. In a PM2.5-mediated lung injury model, AS-IV can decrease serum NF-κB, TNF-α, IL-6, and IL-1β and can also improve the oxidative stress level in BALF, restore the GSH level in the lung tissue, and reduce the iron content in the lung tissue via the Nrf2/SLC7A11/GPX4 axis 52. AS-IV might play a protective role against adriamycin-induced myocardial fibrosis, which may partly be attributed to its antiferroptosis action by enhancing Nrf2 signaling^53^. Tang et al.^54^ also reported that AS-IV inhibited miR-138-5p expression, subsequently increasing Sirt1/Nrf2 activity and cellular antioxidant capacity to alleviate ferroptosis, resulting in decreased cell death, which potentially inhibits the DR pathological process. Nrf2 pathway activity is thought to restore iron homeostasis, limit ROS production, and upregulate SLC7A11 by reducing intracellular iron pools^55^. In addition to being an antioxidant and cytoprotective gene, HO-1 is also a downstream target of Nrf2^56^. As a result, the therapeutic function of AS-IV may be mediated by activating the Nrf2/HO-1 pathway. Future studies should focus on the molecular mechanisms by which AS-IV exerts its neuroprotective effects on ischemic stroke.

## Conclusion

In summary, our research provides evidence that neuroinflammation and ferroptosis are critical cellular modulatory mechanisms that result in EBI following MCAO. We reported the AS-IV-mediated regulation of neuroinflammation and ferroptosis by the Nrf2/HO-1 signaling pathway and offered an innovative idea to examine the biological impacts and mechanisms involved in the antineuroinflammatory, anti-ferroptosis, and neuroprotective effects of AS-IV.

## Data Availability

The data will be available upon request..
